# 418. Possible Risk Factors and Clinical Features of Post-COVID-19 Syndrome in a Veteran Affairs population

**DOI:** 10.1093/ofid/ofad500.488

**Published:** 2023-11-27

**Authors:** Michael D Lum, Monirul I Sajib, Audun Lier

**Affiliations:** Stony Brook University Hospital, Stony Brook, New York; Stony Brook University Hospital, Stony Brook, New York; Stony Brook University Hopstial, Stony Brook, New York

## Abstract

**Background:**

Post-COVID-19 syndrome (PCS) refers to debilitating physical and neuropsychiatric symptoms for at least ≥ 4 weeks following the recovery from COVID-19 infection. The goal of this study is to identify shared epidemiological and clinical features among patients with PCS to identify possible risk factors that can guide studies in the prevention of PCS.

**Methods:**

Chart reviews of patients referred to the post-COVID clinic of Northport VA Medical Center, NY between January 2021 to March 2023 were performed. 57 patients with mild COVID-19 infection without requiring hospitalization that met CDC definition of PCS were included. Cases were reviewed and demographics, comorbidities, treatment/vaccine status, common symptoms reported and baseline inflammatory markers were recorded and analyzed.

**Results:**

The mean age was 56 years, and most patients were between the ages of 46-60 years old (28.1%, table 1). More male patients reported symptoms of PCS compared to females (56.1% vs. 43.9%, table 1). Common comorbidities included pre-existing psychiatric diagnoses (47.4%), obesity (40.4%), hypertension (40.4%), hyperlipidemia (43.9%), and chronic lung disease (36.9%), with an average Charlson comorbidity index of 1.9. Most patients (59.6%) reported only being infected with COVID-19 once (table 1). Most patients (75.4%) did not receive outpatient therapy against COVID-19 and most had only two vaccines against COVID-19 (38.6%, table 2). Predominant symptoms included fatigue (56.1%), brain fog (56.1%), dyspnea on exertion/decreased exercise tolerance (42.1%), and cough (24.6%) (table 3). Of those with values measured, baseline ESR, CRP, ferritin values were normal and a small percentage had elevated D-dimer >150 ng/mL D-DU (table 4).

**Figure:**
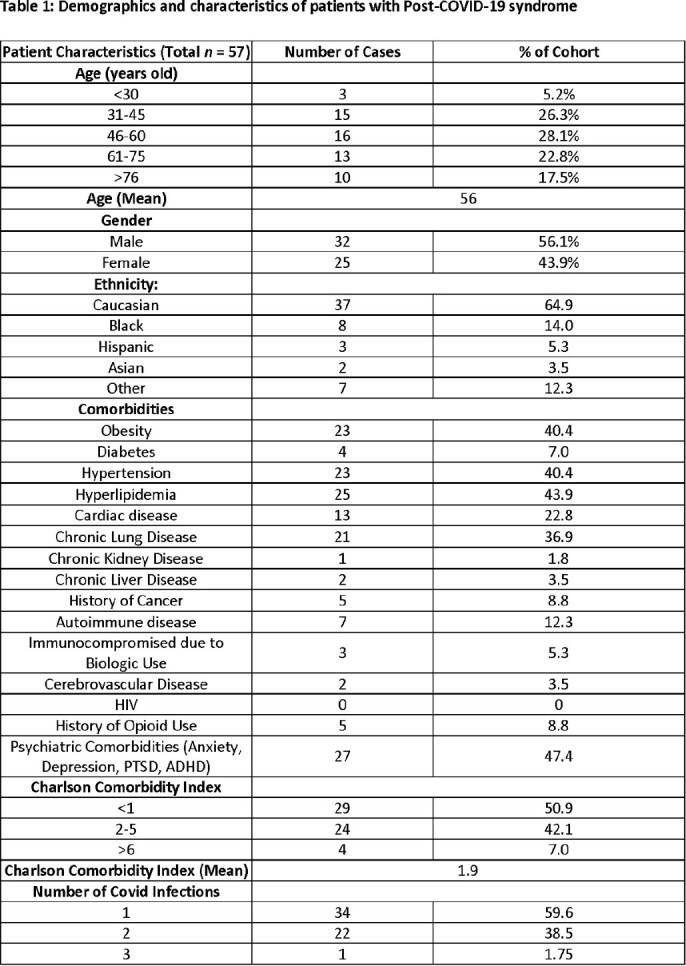

**Figure:**
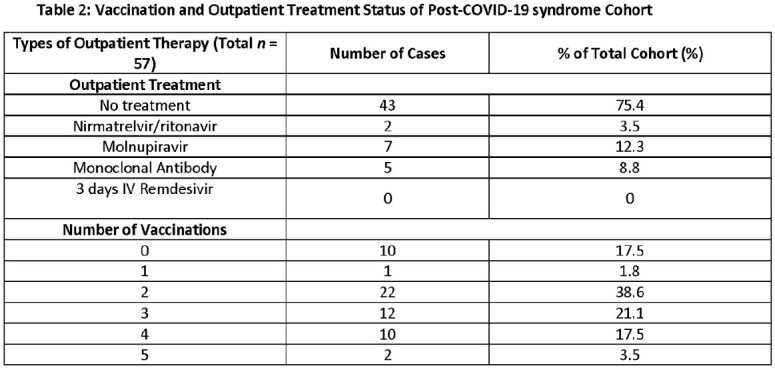

**Figure:**
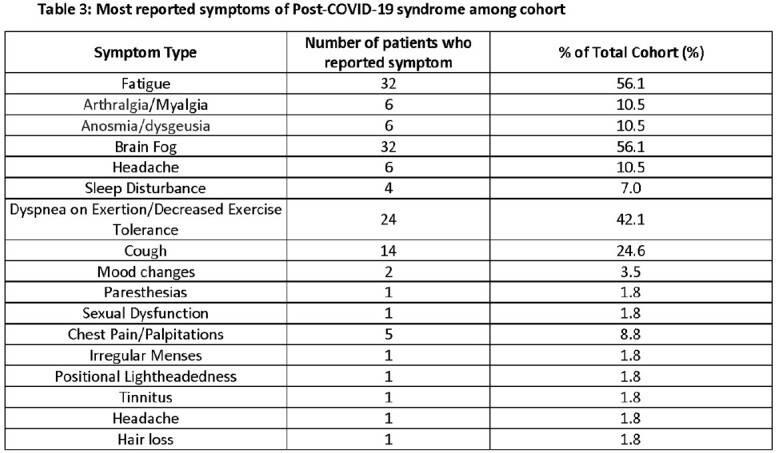

**Conclusion:**

Majority had comorbidities such as hypertension, hyperlipidemia, pre-existing mental health conditions and obesity. Most had only 2 doses of a COVID-19 vaccine and did not receive any outpatient treatment for COVID-19. This suggests that these features may serve as potential risk factors for PCS and further study is needed to elucidate the relationship between these attributes and PCS.

**Disclosures:**

**All Authors**: No reported disclosures

